# Immobilization of *E. coli* expressing γ-glutamyltranspeptidase on its surface for γ-glutamyl compound production

**DOI:** 10.1186/s13568-023-01528-9

**Published:** 2023-03-04

**Authors:** Shintaro Arai, Hideyuki Suzuki

**Affiliations:** Division of Applied Biology, Kyoto Institute of Technology, Goshokaido-Cho, Matsugasaki, Sakyo-Ku, Kyoto, 606-8585 Japan

**Keywords:** γ-glutamyl peptide, Kokumi, γ-glutamyltransferase, γ-glutamylglutamine, Alginate

## Abstract

An *Escherichia coli* strain expressing γ-glutamyltranspeptidase on its extracellular surface using the Met1 to Arg232 fragment of YiaT of *E. coli* as an anchor protein was immobilized with alginate for repeated use. Measurement of γ-glutamyltranspeptidase activity of the immobilized cells was performed repeatedly at pH 8.73 and 37 °C for 10 days using γ-glutamyl-*p*-nitroanilide in the presence of 100 mM CaCl_2_ and 3% NaCl with and without glycylglycine. Even after the 10th day, the enzyme activity did not decrease from the initial levels. The production of γ-glutamylglutamine from glutamine using the immobilized cells was performed repeatedly at pH 10.5 and 37 °C for 10 days in the presence of 250 mM glutamine, 100 mM CaCl_2_, and 3% NaCl. Sixty-four % of glutamine was converted to γ-glutamylglutamine in the first cycle. While repeating the production 10 times, the surface of the beads gradually became covered with white precipitate, and the conversion efficiency gradually decreased, but 72% of the initial value still remained even at the 10th measurement.

## Introduction

**Fig. 1:**
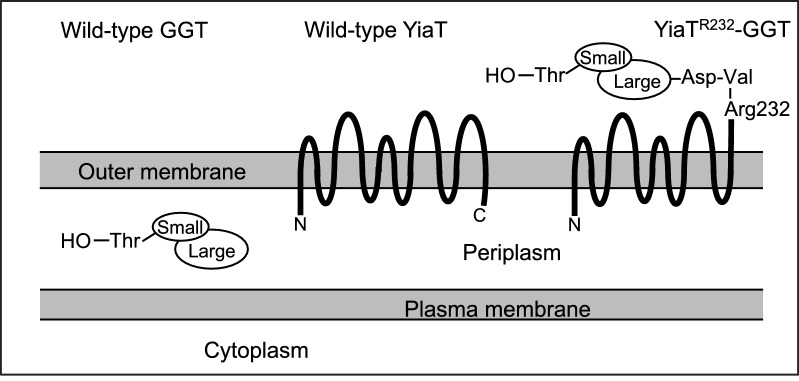
Wild-type and cell-surface expressing forms of *E. coli* GGT. Arg232 is the residue in the fifth extracellular loop of YiaT, but the C-terminal of the wild-type YiaT protein is in the periplasm (Han and Lee 2015). By connecting GGT to the Arg232 residue of YiaT, GGT was expressed on the extracellular surface of *E. coli* cells (Suzuki and Sasabu 2023)

The γ-glutamyl linkage is formed between the γ-carboxyl group of glutamic acid and the amino group of an amino acid or peptide. The most well-known γ-glutamyl peptide in nature is glutathione (γ-Glu-Cys-Gly). Including glutathione, several γ-glutamyl compounds were reported as kokumi substances (Ueda et al. 1990, 1997; Dunkel et al. 2007; Toelstede et al. 2009; Toelstede and Hofmann 2009; Ohsu et al. 2010; Kuroda et al. 2012; Ho and Suzuki 2013; Miyamura et al. 2014; Hillmann et al. 2016; Hillmann and Hofmann 2016; Phewpan et al. 2020). A small amount of kokumi substances can increase the thickness, continuity, and mouthfullness of various dishes (Ueda et al. 1990). We have reported that purified bacterial γ-glutamyltranspeptidase (GGT; EC 2.3.2.2) is an excellent catalyst for the enzymatic production of γ-glutamyl compounds (Suzuki et al. 2007, 2020; Suzuki 2019, 2021). This enzyme catalyzes two reactions. One is a reaction that hydrolyzes the γ-glutamyl linkage of γ-glutamyl compounds, a hydrolysis reaction. The other is a reaction that transfers the γ-glutamyl moiety from γ-glutamyl compounds to other amino acids or peptides to form new γ-glutamyl compounds, a γ-glutamyl transpeptidation reaction (Tate and Meister 1981; Taniguchi and Ikeda 1998). These reactions depend on pH, and the hydrolysis and transpeptidation reactions mainly proceed under acidic and basic conditions, respectively. Therefore, GGT can be used selectively to catalyze the transpeptidation reaction by adjusting the pH of the reaction mixture to basic conditions. *E. coli* GGT has broad substrate specificity against γ-glutamyl acceptors (Suzuki et al. 1986). For example, we developed the enzymatic method to produce γ-Glu-Val-Gly, one of the kokumi γ-glutamyl peptides, using purified *E. coli* GGT (Fukao and Suzuki 2021). Among enzymatic production of γ-glutamyl peptides by purified *E. coli* GGT that we have developed, γ-Glu-Gln production is one of the most successful examples in terms of conversion (El Sayed et al 2010). In this case, Gln is the only substrate and we can simplify the reaction condition. The transpeptidation activity of *E. coli* GGT purified from the periplasm is much higher than the hydrolysis activity under basic pH. However, the activity of whole cells containing GGT in the periplasm was mostly hydrolysis activity, even under basic pH. For this reason, it was necessary to purify GGT from *E. coli* cells to use for synthesizing γ-glutamyl compounds, and it takes time and labor to purify the enzyme. We developed a method to express GGT on the extracellular surface of *E. coli* cells using the Met1 to Arg232 fragment of *E. coli* YiaT as an anchor protein (Fig. 1). These cells synthesized 8 mM γ-Glu-Val-Gly from 20 mM Gln and 100 mM Val-Gly in 2 h without any purification treatment of GGT (Suzuki and Sasabu 2023). Still, because of their size, once the reaction is finished, it is difficult to recover the bacterial cells from the reaction mixture and use them for the next production cycle. Immobilization is one of the methods developed for the repeated use of enzymes and cells (Basso and Serban 2019; Chibata 1986). Several papers have reported the immobilization of bacterial GGT, but the methods still required the purification of GGT (Hung et al. 2008; Chen et al. 2013; Juang et al. 2014; Wang et al. 2014; Bindal and Gupta 2016; Ni et al. 2017). Furthermore, *E. coli* cells harboring the pET vector with the *Pseudomonas nitroreducens ggt* gene were immobilized (Phumsombat et al. 2020). GGT of *P. nitroreducens* exhibits much higher hydrolysis activity than transpeptidation activity, even if it was purified, which is different from other GGTs. In addition, it was reported that there was no significant difference in the theanine (γ-glutamylethylamide) productivity of wild-type *P. nitroreducens* and *E. coli* expressing *ggt* gene of *P. nitroreducens* from the T7 promoter, using the cell-free extracts and whole cells of each (Phumsombat et al. 2020). In another report, *E. coli* cells expressing *E. coli* GGT from T7 promoter were immobilized in alginate beads and used for theanine production. In that case, only 12.8% of the initial yield was obtained on the tenth cycle of repeated use of the immobilized cells (Zhang et al. 2010). However, the cellular localization of recombinant GGTs was not reported in either case.

γ-Glu-Gln is one of the kokumi γ-glutamyl dipeptides found in Gouda cheese (Toelstede et al. 2009), Parmesan cheese (Hillmann et al. 2016; Hillmann and Hofmann 2016), Shropshire Blue, and other cheeses (Toelstede and Hofmann 2009). Furthermore, though glutamine is not essential for healthy human beings, it may be useful in certain cases (Lacey and Wilmore 1990; Houndijk et al. 1998; Thomas and Balasubramanian 2003). For example, oral glutamine administration in athletes after intense exercise decreases the risk of infections caused by immunosuppression (Castell and Newsholme 1997). Despite the positive effects of glutamine, glutamine is not included in commercially available aqueous supplements or in infusion solutions used in the medical field because it is unstable in aqueous solutions. Therefore, we developed a method to produce γ-Glu-Gln using purified *E. coli* GGT, and showed that γ-Glu-Gln is stable in an aqueous solution (El Sayed et al. 2010).

In this study, we immobilized the *E. coli* cells expressing GGT on the extracellular surface in alginate beads and confirmed its usability as a catalyst to repeatedly produce a γ-glutamyl compound.

## Materials and methods

### Chemicals

Sodium alginate and γ-Glu-Gln were purchased from Fujifilm Wako Chemicals (Osaka, Japan) and Kokusan Chemical (Tokyo, Japan), respectively. Other amino acids and reagents were purchased from Nacalai Tesque (Kyoto, Japan).

### Cell cultivation

*Escherichia coli* K-12 strain SH2161 (pSH2074/SK123) was used in this study. The plasmid pSH2074 expresses YiaT^R232^-GGT fusion protein from T5 promoter. This plasmid is a pBR322-based plasmid containing *E. coli yiaT* gene up to Arg232 residue (Suzuki and Sasabu 2023) as an anchor protein, fused with the large and small subunits coding regions without the signal peptide of *E. coli ggt* gene. The genotype of strain SK123 is F^−^ *rph-1* Δ*ggt-2* Δ*puuD*::FRT. Therefore, γ-glutamyl-*p*-nitroanilide (γ-G*p*NA) cleaving activity only depends on the *ggt* gene on pSH2074. The strain was grown at 37 °C in 5 mL of Luria–Bertani (LB Miller) medium with 100 µg/mL of ampicillin overnight. LB medium (20 mL) with 100 µg/mL of ampicillin was inoculated with the preculture to an initial optical density at 600 nm (OD_600_) of 0.05 and incubated at 37 °C with reciprocal shaking at 120 rpm. After 2 h, a final concentration of 0.5 mM isopropyl-β-d-1-thiogalactopyranoside was added to the medium to induce the expression of GGT. After further incubation at 20 °C for 24 h, the cells were harvested by centrifugation at 5000×*g* for 5 min at 4 °C. The pellet was washed with 10 mL of 200 mM Tris-HCl (pH 7.4), collected by centrifugation, and resuspended with 200 mM Tris-HCl (pH 7.4). The resuspended solution was used for the experiments.

### Cell immobilization

Two percent sodium alginate was used for immobilization as described previously (Zhang et al. 2010). First, 4.5 mL of autoclaved sodium alginate solution and 0.5 mL of the resuspended solution of strain SH2161 were mixed well. Then, the mixture was dropped into a sterile 200 mM CaCl_2_ solution to make beads. The beads were kept still at 4 °C for 1 h to cure. They were washed three times with 5 mL of sterile 20 mM Tris-HCl (pH 7.4) containing 100 mM CaCl_2_. Then, the beads were used for the experiments. The diameter of the beads was 4 mm. The OD_600_ of the cells in the mixture of sodium alginate solution and resuspended cell solution was adjusted to 0.5 as a baseline, and it was adjusted to 1.0 when γ-Glu-Gln production was performed.

### Measurement of GGT activity

GGT activity was determined spectrophotometrically by measuring the *p*-nitroaniline released from γ-G*p*NA (Suzuki et al. 1986). The assay solution that contained 0.5 mM γ-G*p*NA, 50 mM Tris-HCl (pH 8.73), 100 mM CaCl_2_, 60 mM Gly-Gly, and 0 or 3% NaCl was used. Since Gly-Gly solution is acidic, the pH of the Gly-Gly stock solution was adjusted to 8.73 beforehand. pH 8.73 is the optimal pH of the transpeptidation reaction of *E. coli* GGT using Gly-Gly as a γ-glutamyl acceptor. The hydrolysis activity was measured using the assay solution without Gly-Gly. The amount of released *p*-nitroaniline was calculated from the change of absorbance at 410 nm using the molar extinction coefficient. The transpeptidation activity was defined as the activity value calculated by subtracting the hydrolysis activity from the activity value obtained from the reaction solution with Gly-Gly, and this value represents the ability to produce γ-glutamyl compounds. One unit of the enzyme was defined as the amount of the enzyme that released 1 μmol of *p*-nitroaniline per minute from γ-G*p*NA.

The reaction was started by the addition of 1.2 mL of assay solution, preincubated at 37 °C, to 4 beads. The reaction proceeded at 37 °C on a rotator at 50 rpm for 20 min. After the beads were separated, the reactions were terminated by the addition of the twofold volume of 3.5 N acetic acid to the supernatants. The recovered beads were washed to remove residuals as follows. The beads were incubated in 7 mL of 20 mM Tris-HCl (pH 7.4) with 100 mM CaCl_2_ for 10 min at 37 °C. Then, the beads were washed twice with 5.0 mL of the same buffer. The total activity was measured with the assay solution containing Gly-Gly. After the beads were washed, the hydrolysis activity was measured for the assay solution without Gly-Gly using the same beads.

### Repeated synthesis of γ-Glu-Gln

The reaction solution consisting of 250 mM Gln, 100 mM CaCl_2_, 3% NaCl, and pH 10.5 was prepared. The reaction was started by the addition of 1.2 mL of the reaction solution, preincubated at 37 °C, to 4 beads. The mixtures were incubated at 37 °C for 22 h to produce γ-Glu-Gln. After the beads were separated, the reaction solution was boiled for 5 min to terminate the reaction. Then, the solution was passed through the Millex-LH Syringe Driven Filter Unit (Millipore; Billerica, MA) and stored at − 25 °C until high-performance liquid chromatography (HPLC) analysis. After each reaction, the beads were washed as described above and used for the next reaction.

### Measurement of γ-Glu-Gln

The concentration of γ-Glu-Gln in the reaction solution was measured using an HPLC instrument (model LC-20A; Shimadzu; Kyoto, Japan) with a fluorescence detector (model RF-10A_XL_; Shimadzu) and equipped with a Shim-pack Amino-Na column (Shimadzu) using *o*-phthalaldehyde as the derivatization reagent, as described previously (Suzuki et al. 2003). The retention time of γ-Glu-Gln was 6.6 min under our condition.

### Statistical analysis

We performed three independent reactions and the average of the three results are shown in the “Results” and “Discussion” with standard deviations calculated by STDEV function in Excel.

## Results

### Effect of immobilization on GGT activity

**Fig. 2:**
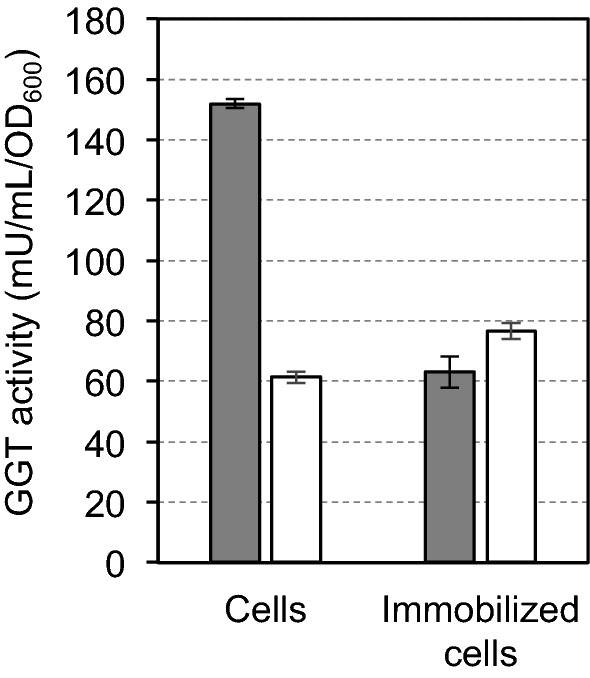
Comparison of transpeptidation (gray bar) and hydrolysis (white bar) activities between the whole cells and the immobilized cells. Enzyme activity was assayed with 0.5 mM γ-G*p*NA, 50 mM Tris-HCl (pH 8.73), and 100 mM CaCl_2_, with and without 60 mM Gly-Gly. The reaction was conducted at 37 °C for 20 min. Values are expressed as the averages of three samples ± standard deviations

The GGT activities between SH2161 cells and its immobilized cells were compared (Fig. 2). The hydrolysis activity (white bars) increased from 61.4 to 76.6 mU/mL/OD_600_, while the transpeptidation activity (gray bars) decreased from 151.9 to 63.1 mU/mL/OD_600_ due to immobilization.

### Effect of the addition of NaCl

**Fig. 3:**
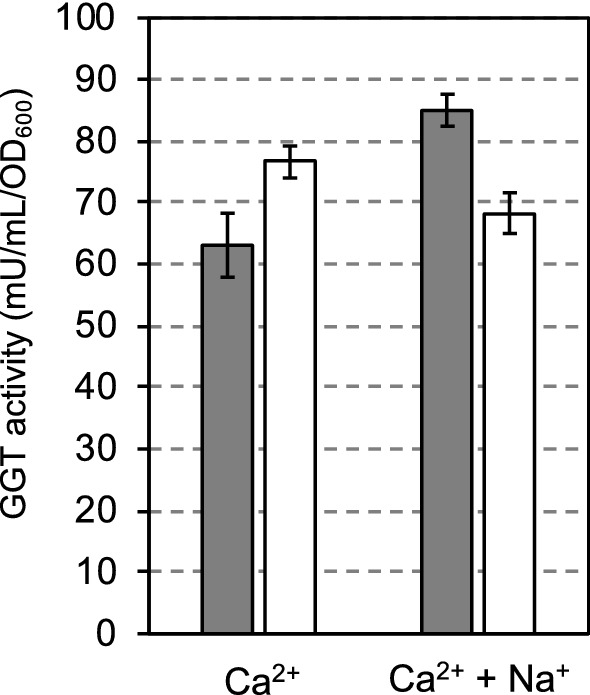
The transpeptidation (gray bar) and the hydrolysis (white bar) activities of the immobilized cells of SH2161 expressing GGT on the extracellular surface were assayed using 100 mM CaCl_2_, with and without 3% NaCl. Values are expressed as the averages of three samples ± standard deviations. The activity without NaCl is taken from Fig. 2

As shown in Fig. 2, the transpeptidation activity of GGT and the ratio of transpeptidation activity to hydrolysis activity decreased by immobilizing the cells, although the transpeptidation activity remained. Previously, we reported that the transpeptidase activity of purified *E. coli* GGT was activated strongly by the addition of NaCl, and several monovalent (Li^+^, K^+^, Rb^+^, Cs^+^) and divalent cations (Mg^2+^, Mn^2+^) also activated transpeptidation activity (Suzuki et al. 2007). Thus, the effect of the addition of 3% NaCl to the assay solutions was investigated. As shown in Fig. 3, the transpeptidation activity increased and the hydrolysis activity decreased with the addition of 3% NaCl.

### Reusability of the immobilized cells

**Fig. 4:**
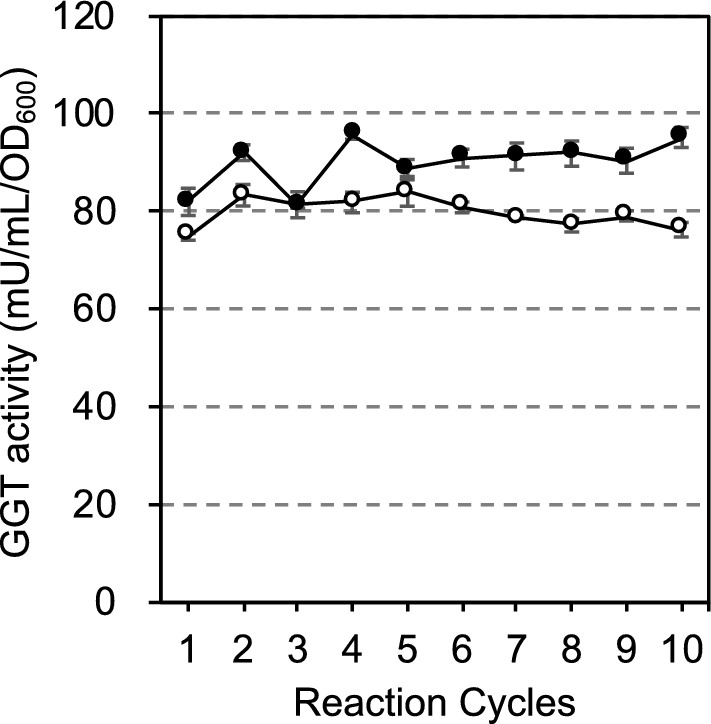
Determining the reusability of the immobilized cells of SH2161. The transpeptidation (black circle) and the hydrolysis (white circle) activities were measured in the presence and absence of Gly-Gly at pH 8.73 for 20 min with 4 beads in the presence of 100 mM CaCl_2_ and 3% NaCl. After the reaction, the beads were washed and then incubated in 1.2 mL of 20 mM Tris-HCl (pH 7.4) with 100 mM CaCl_2_ at 37 °C for 22 h. This cycle was repeated 10 times. Values are expressed as the averages of three samples ± standard deviations

To confirm the reusability of the immobilized SH2161 cells, the enzyme activity of GGT was measured 10 times. As shown in Fig. 4, the GGT activities of immobilized cells of SH2161 were stably maintained even at the tenth measurement, after 10 days of the initial measurement.

### Repeated production of γ-Glu-Gln using the immobilized cells of strain SH2161

γ-Glu-Gln is not only one of the kokumi γ-glutamyl peptides, but can also be applicable to infusion solutions. Moreover, γ-Glu-Gln production is one of our successful examples of γ-glutamyl compound production using purified *E. coli* GGT. Therefore, we investigated the repeated production of γ-Glu-Gln as an example of γ-glutamyl compound production using the immobilized cells of SH2161.

**Fig. 5:**
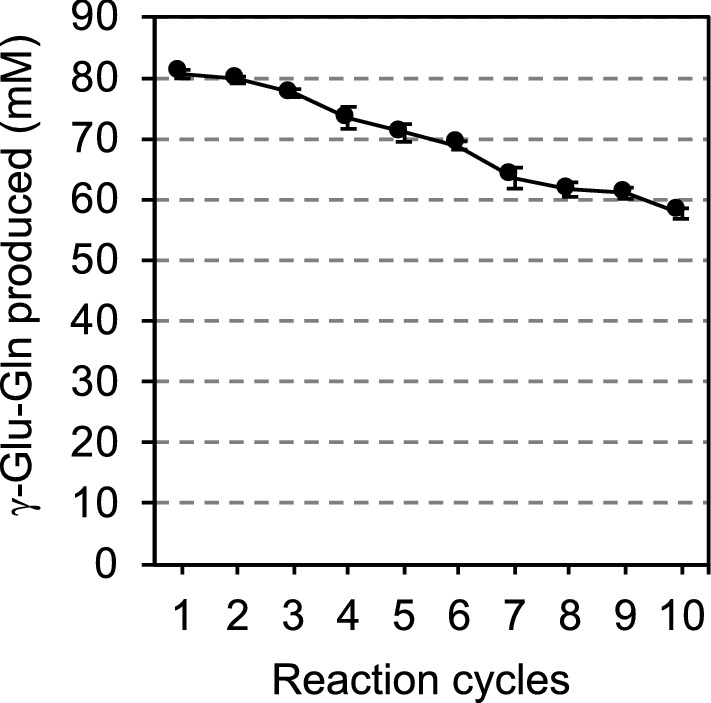
Production of γ-Glu-Gln using the immobilized cells of SH2161 during each reaction cycle in 22 h. The reaction was performed in 250 mM Gln, 100 mM CaCl_2_, and 3% NaCl at pH 10.5, at 37 °C for 22 h with 4 beads. After the reaction, the beads were washed using 1.2 mL of 20 mM Tris-HCl (pH 7.4) with 100 mM CaCl_2_. This cycle was repeated 10 times. Values are expressed as the averages of three samples ± standard deviations

Only γ-Glu-Gln was produced as a γ-glutamyl product with our reaction condition. The decrease in γ-Glu-Gln synthesis was observed with the increasing number of reaction cycles (Fig. 5). Still, the γ-Glu-Gln yield of the tenth cycle was about 72% of the first cycle.

## Discussion

As we reported previously (Suzuki and Sasabu 2023), SH2161 cells express GGT on the extracellular surface of the cells using YiaT^R232^ as an anchor protein and these cells were used as a catalyst to produce a γ-glutamyl compound. However, bacterial cells are not appropriate for repeated use. Therefore, we immobilized SH2161 cells in alginate beads, and their activity was compared with that of the free cells. As we used CaCl_2_ to immobilize the cells in alginate beads, we compared GGT activities in the presence of 100 mM CaCl_2_. By immobilizing the cells, the transpeptidation activity decreased (Fig. 2). The result suggests that even though GGT is expressed on the extracellular surface of the cells, its surroundings in the beads of alginate are not as free as in the solution. By the addition of 3% NaCl, the ratio of transpeptidation activity to hydrolysis activity became greater than 1 (Fig. 3). This encouraged us to use immobilized SH2161 cells for γ-glutamyl compound production. Although the mechanism of the effect of NaCl is not clear, it may be because the transpeptidation activity was activated as reported previously (Suzuki et al. 2007) or the extracellular expression of GGT was enhanced (Suzuki and Sasabu 2023).

As GGT activities of the immobilized cells of SH2161 measured by γ-G*p*NA and Gly-Gly were stably maintained over 10 days (Fig. 4), the immobilized cells were subjected to the repeated production of γ-Glu-Gln. Although the yield of γ-Glu-Gln gradually decreased and at the tenth cycle it was about 72% of the first cycle (Fig. 5), this was much better than the previous report in which the theanine yield in the tenth cycle decreased to 12.8% of the first cycle using immobilized *E. coli* cells expressing *E. coli* GGT from the T7 promoter (Zhang et al. 2010). We realized that *E. coli* cells expressed GGT from strong T5 promoter have GGT on the extracellular surface of the cells just after harvest, but thereafter GGT was released from the cells in the absence of NaCl (Suzuki and Sasabu 2023). Since the size of the cells and that of GGT are very much different, it is difficult to immobilize both at a time. It has been reported that enzymes immobilized in calcium alginate beads tend to leak (Labus et al. 2020). This may be the reason why the recovery of theanine decreased by the repeated use of the immobilized cells in the case of Zhang et al. (2010). On the other hand, our strain was able to maintain its activity for a long time by fixing GGT on the extracellular surface of the cells using an anchor protein. In the experiment of Fig. 5, however, the immobilized cells were used for γ-Glu-Gln production at pH 10.5 for 22 h in the presence of 100 mM CaCl_2_ repeatedly, and the surface of the beads was gradually covered with a white precipitate which may be Ca(OH)_2_. The precipitate on the surface of the beads may prevent the substrate from migrating into the beads and this may have attenuated the γ-Glu-Gln production over time. Our beads cracked after 4 cycles of γ-Glu-Gln production without the addition of CaCl_2_ to the reaction mixture. Thus, the addition of CaCl_2_ not only activated GGT (Suzuki, et al. 2007) but prevented cracking. In the experiment of Fig. 4, the enzymatic reaction was performed at pH 8.73 for 20 min and the surface of the beads was not covered with precipitate. We think this is why the enzymatic activity remained well even at the 10th cycle in Fig. 4. As we reported previously (El Sayed et al. 2010), the efficiency of γ-Glu-Gln production was very much affected by the reaction pH. Considering the total production of 10 times, we decided that it would be better to produce at pH 10.5.

In this study, the cells of strain SH2126 expressing GGT on the extracellular surface of the cells with an anchor protein were successfully immobilized with alginate, and the GGT activity remained well after repeated use. Therefore, these immobilized beads were useful for the production of a γ-glutamyl compound.

## Data Availability

Some data that support the findings of this study are available from the corresponding author upon reasonable request. The strains and plasmids used in this work can be shared by exchanging MTA.

## Abbreviations

GGT: γ-Glutamyltranspeptidase
γ-G*p*NA: γ-L-glutamyl-*p*-nitroanilide
γ-Glu-Gln: γ-Glutamylglutamine
HPLC: High-performance liquid chromatography
γ-Glu-Val-Gly: γ-Glutamylvalylglycine
OD_600_: Optical density at 600 nm
